# Interaction between Smoking and HLA-DRB1*04 Gene Is Associated with a High Cardiovascular Risk in Brazilian Amazon Patients with Rheumatoid Arthritis

**DOI:** 10.1371/journal.pone.0041588

**Published:** 2012-08-13

**Authors:** Narjara de Oliveira Boechat, Mauricio Morish Ogusku, Antonio Luiz Boechat, Aya Sadahiro

**Affiliations:** 1 Programa de Pós-Graduação em Imunologia Básica e Aplicada, Instituto de Ciências Biológicas, Universidade Federal do Amazonas, Manaus, Brasil; 2 Clínica de Artrite Reumatoide, Hospital Geral Adriano Jorge, Manaus, Brasil; 3 Laboratório de Micobacteriologia, Instituto Nacional de Pesquisas da Amazônia (INPA), Manaus, Amazonas, Brasil; The University of Texas M. D. Anderson Cancer Center, United States of America

## Abstract

**Background:**

Rheumatoid Arthritis (RA) is an autoimmune disease characterized by chronic inflammation of the joints that affects approximately 1% of the population worldwide. The *HLA-DRB1* gene locus plays a major role in genetic susceptibility to RA, a condition that has been associated with a high cardiovascular morbidity and mortality in many studies.

**Methodology/Principal Findings:**

The aim of this work was to investigate which types of HLA class II genes are associated with RA in patients from the Brazilian Amazon and their influence on high cardiovascular risk status in this population. For this purpose, a case-control study was carried out with a total of 350 non-Indian individuals made up of a cohort of 132 consecutive RA sufferers and 218 healthy controls. A χ^2^ test showed that HLADRB1*04 (*p*<0.0016; OR = 1.89; 95% CI = 1.29–2.79) and HLADRB1*10 (*p* = 0.0377; OR = 3.81; 95% CI = 1.16–12.50) are the major *HLA* genes associated with susceptibility to RA. A logistic regression model also showed that the interaction between HLADRB1*04 (*p* = 0.027; OR = 6.02; 95% CI = 1.21–29.7), age (*p* = 0.0001; OR = 1.26; 95% CI = 1.13–1.39) and smoking (*p* = 0.0001; OR = 23.6; 95% CI = 4.25–32.1) is associated with a probability of a high cardiovascular risk status at an early age.

**Conclusions/Significance:**

The results of this study show for the first time that HLA class II type is associated with RA in Brazilian Amazon populations and that a specific interaction between the HLA-DRB1*04 gene and smoking is associated with a high cardiovascular risk status, as initially reported in the European population. This study therefore contributes to an understanding of gene-environment interactions in RA patients.

## Introduction

Rheumatoid arthritis (RA) is an autoimmune disease characterized by chronic inflammation of the synovial membranes of the joints [1]. Its incidence varies between 0.4% and 1.9%worldwide and 0.2% to 1.0% in the Brazilian population [2], [3]. An increased prevalence of heart and coronary diseases and a high rate of cardiovascular (CV) mortality were observed in patients with RA [4]. However, the underlying mechanism behind the high prevalence of CV morbidity in RA is not completely understood [5], and there is little information on the influence that genetic factors may have on mortality in RA. It has been speculated that genes that play an important role in the development and progression of RA may also play a role in comorbidity and mortality in this disease [6].

Studies have proved that classical CV risk factors, such as diabetes mellitus, hypertension, hypercholesterolemia, smoking and obesity, have similar effects in both the general and RA population, but the CV risk profile of RA patients has not been thoroughly studied. Previous studies of cardiovascular disease in RA concentrated on fatal CV events [7] and neither attempted to estimate the relative contribution of CV risk factors and clinical manifestations of RA nor examined how these two aspects interact with each other [5]. In one study, the increased risk of cardiovascular events in RA patients was found to be independent of traditional cardiovascular risk factors [7]. Thus, the search for other predictors of cardiovascular disease in this population is of great importance.

Environmental and genetic factors play an important role in the development of RA, and the genetic component accounts for up to 60% of susceptibility to the disease [8]. The largest genetic contribution is provided by the major histocompatibility complex (MHC) Class II genes, in particular *HLA-DRB1* alleles, several of which (HLA-DRB1*0401, *0404, *0405, *0408, *0101, *0102,*1001 and *1402) are associated with susceptibility to RA. They encode a conserved amino acid sequence (QKRAA, QRRAA or RRRAA) called the shared epitope (SE) at positions 70–74 in the third hypervariable region of the HLA-DRβ1 molecule [9].There is preliminary evidence that the increased cardiovascular risk in patients with RA can be, at least partly, associated with polymorphism in the *HLA-DRB1* locus [10], [11]. Shared epitope (SE) HLA-DRB1*04 alleles have already been associated with endothelial dysfunction and may lead to an increased CV risk [6], [12]. However, to our knowledge, no studies have been carried out to investigate the influence of HLA Class II on RA patients in the Brazilian Amazon or the clinical or biological implications of these genes for this population. The aim of this study was therefore to investigate the CV risk profile associated with HLA class II in RA patients in the Brazilian Amazon.

## Methods

### Objectives

This study aimed to evaluate the association between HLA Class II and RA in individuals from the Brazilian Amazon and the influence of this association on high cardiovascular risk status in these individuals.

### Participants

The study population consisted of 350 non-Indian individuals from the Brazilian Amazon, made up of a cohort of 132 consecutive RA sufferers and 218 controls without any apparent diseases. The RA sufferers had been referred to the Rheumatoid Arthritis Clinic in the Adriano Jorge General Hospital, Manaus, Brazil and were selected in accordance with the American College of Rheumatology revised criteria for the classification of RA (1987) and the 2010 American College of Rheumatology/European League Against Rheumatism classification criteria for Rheumatoid Arthritis [13], [14]. Controls were randomly selected by inviting people who lived in the same area as the patients to take part in the study. Controls were considered healthy if their medical history did not reveal any chronic diseases, endemic infectious diseases or autoimmune diseases and their physical examination and blood tests (e.g. glucose) failed to show otherwise. Any individual with one or more of these conditions was excluded from the control sample. For individuals who satisfied the medical history requirement, 5 mL of blood sample was collected and genomic DNA extracted, as was done for the patients. The DNA samples were registered and stored in a control DNA bank. When this study began, a number was selected at random from the register and the corresponding control and appropriate number of sequentially numbered controls were taken from the DNA bank, matching age and sex as required.

### Description of Procedures

The following information was gathered from the patients' medical records and collated on a form specifically prepared for this purpose: disease duration; age at onset; results of rheumatoid factor (RF), erythrocyte sedimentation rate (ESR), C-reactive protein (CRP), complete blood count, kidney and liver function, blood glucose and lipid profile tests at the time of assessment; disease modifying antirheumatic drugs (DMARDs) being used; and use of anti-TNF drugs. Weight, height, a history of smoking and other cardiovascular risk factors were also recorded. Patients were classified as having either only joint involvement or systemic involvement, the latter being considered to be present when at least one of the following clinical findings was detected: subcutaneous nodules, lung fibrosis confirmed by high-resolution computed tomography of the thorax; pleural effusion confirmed by chest X-ray; pericardial effusion evidenced by echocardiography; Felty's syndrome (white blood cell count <2×10^6^ cells, splenomegaly); cutaneous vasculitis (histopathological evidence of leukocytoclastic vasculitis); peripheral non-entrapment neuropathy (confirmed by electroneuromyography); Sjögren's syndrome (confirmed by the Schirmer's test, biopsy of minor salivary glands or MRI of salivary glands). Disease activity was determined according to the Disease Activity Score for 28 joints (DAS28) [15] as well as the Health Assessment Questionnaire (HAQ) validated for the Portuguese language [16], [17]. Serious cases were considered to be those with at least one of the systemic manifestations previously mentioned and/or use of immunotherapy. The Framingham Coronary Risk Score was determined for each patient based on the individual's clinical information and by applying a factor of 1.5 for those patients who had at least two of the following: a) extra-articular disease; b) more than 10 years of disease; or c) positive rheumatoid factor or positive anti-citrullinated protein/peptide antibodies (ACPA). Individuals with RA have a relative risk of death from cardiovascular disease approximately 1.5 times greater than individuals without RA [18]. Hence, the values calculated for the Framingham cardiovascular risk score [19] for the severe cases described in the present study must be corrected by multiplying them by a factor of 1.5, as recommended by EULAR (The European League against Rheumatism) [20].

### Ethics

This study was approved by the Federal University of Amazonas Human Research Ethics Committee (reference number 0028.0.115.000-0). All the study participants provided written informed consent in accordance with the Declaration of Helsinki both for the *HLA Class II* genotyping and for access to their clinical data before they enrolled in the study.

### Extraction of genomic DNA and generic HLA Class II genotyping

DNA was extracted using 5 mL of peripheral blood and a rapid technique using trimethyl ammonium bromide salts (DTAB/CTAB) [21]. Class II HLA typing was performed by polymerase chain reaction (PCR) using sequence-specific Primers (Micro SSP™ Generic HLA class II DNA typing tray, One Lambda Inc), following the manufacturer's instructions. The amplified DNA fragments were separated by electrophoresis on a 2.5% agarose gel and visualized by staining with SYBRGreen and exposure to a blue light transilluminator. DNALMT version 3.81 from One Lambda was used for the final typing of *HLA* alleles.

### Statistical methods

Descriptive statistics were used to characterize the profile of RA patients who were selected for the study. The comparison of means was carried out with a two-tailed Student t-test when a normal distribution was observed in a Kolmogorov-Smirnov normality test. To test for an association between HLA Class II genes and RA, a univariate chi-square test with p<0.05 and a 95% confidence interval was used. Based on the types of class II HLA identified in individuals with RA, a stepwise forward logistic regression model was built to analyze the influence of these genes on susceptibility to RA. The relationship between high cardiovascular risk and the types of HLAclass II found to be associated with RA was investigated using a second logistic regression model and a Framingham Coronary Risk score of over 20% (high cardiovascular risk) for each patient as the dependent variable. A goodness-of-fit test was performed using Hosmer-Lemeshow tests. Receiver Operating Characteristic (ROC) curves were plotted and the corresponding Areas Under the Curves (AUCs) calculated to check for the accuracy of each logistic regression model. Statistical analysis was performed using the R Statistical package.

## Results

### Subjects

The study population consisted of one hundred thirty-two consecutive RA patients with a mean age at onset of RA symptoms of 42.4 years and mean disease duration of 4.85 years. Of these, 98 (74.2%) were RF seropositive and 34 had extra-articular involvement in which Sjögren Syndrome was the most prevalent systemic manifestation. No statistical differences were observed for mean age and sex distribution of RA patients and controls. Other important demographic information for the study population is shown in Table 1.

**Table 1:** Characteristics of 132 RA patients from the Brazilian Amazon.

RA features	132 RA cases	218 Control Individuals
Female/Male, n (%)	119 (90.2%)/13 (9.8%) *	196 (89.9%)/22 (10.1%) *
Age, mean (SD)	48.1 (12.3) **	50.5 (9.97) **
Positive Rheumatoid Factor, n (%)	98 (74.2%)	-
Age at onset, mean (SD)	42.4 (±13.2)	-
Disease Duration in years, mean (SD)	4.9 (±3.5)	-
Early Rheumatoid Arthritis	43 (32.6%)	-
DAS28, mean (SD)	4.51 (±1.59)	-
HAQ, mean (SD)	1.68 (±0.9)	
Extra-articular manifestations, n (%)	34 (25.7%)	-
Sjögren Syndrome, %	15.9%	
Rheumatoid Nodules, %	6.4%	
Peripheral Neuropathy, %	1.5%	
Pulmonary Fibrosis, %	1.5%	
DMARDS, n (%)	92 (69.7%)	-
Use of Anti-TNF, n (%)	40 (30.3%)	-
Years of Education, mean (SD)	9.4 (±4.6)	-
Higher Education, n (%)	4 (3.0%)	-
**Traditional Factors for CVD**		-
Current or Past Smoking, n (%)	44 (33.3%)	-
Hypertension, n (%)	16 (11.4%)	-
Dyslipidemia, n (%)	81 (60.4%)	-
Diabetes, n (%)	4 (3%)	-

* *p* = 0.360;

** *p* = 0.941.

### Generic HLA Class II genotyping and its association with RA

Table 2 contains the ClassII HLA profiles of the RA patients studied, which show that HLA-DRB*04 (*p*<0.0016; OR = 1.89; 95% CI = 1.29–2.79), HLA-DRB1*10 and DQB1*0302 (*p* = 0.0027; OR = 1.90; 95% CI = 1.30–2.90) are associated with risk factors for RA. HLA-DRB1*16 (*p* = 0.029; OR = 0.41; 95% CI = 0.2–0.88) and HLA-DQB1*0303 are associated with protection against RA.HLA-DQB1*05 and *HLA-DRB5* also show a trend towards an association with RA risk factors but without statistical significance. As can be seen in Table 3, none of these genes appears to exert any influence on the clinical features of RA.

**Table 2:** Analysis of ClassII HLA and its association with rheumatoid arthritis*.

Generic HLA Class II	RA patients	Controls	*p*	OR	95% Confidence Interval
	(n = 132)	(n = 218)			
*DRB1* * *01*	29	31	0.1019	1.61	0.95–2.74
*DRB1* * *0301*	13	25	0.7748	0.8515	0.43–1.70
*DRB1* * *0302*	2	6	0.7044	0.5471	0.11–2.73
*DRB1* * *04*	64	63	0.0016	1.8946	1.23–2.79
*DRB1* * *07*	17	44	0.1279	0.6132	0.34–1.10
*DRB1* * *08*	21	51	0.1466	0.6524	0.38–1.11
*DRB1* * *09*	6	12	0.8869	0.8217	0.30–2.21
*DRB1* * *10*	9	4	0.0377	3.8118	1.16–12.50
*DRB1* * *11*	14	40	0.0865	0.5544	0.30–1.03
*DRB1* * *12*	1	2	0.6599	0.8251	–
*DRB1* * *13*	25	44	0.8912	0.9319	0.56–1.56
*DRB1* * *14*	20	30	0.8457	1.1093	0.61–2.00
*DRB1* * *15*	20	30	0.8457	2.1093	0.61–2.00
*DRB1* * *16*	9	34	0.0291	0.4173	0.20–0.88
*DRB3* *	66	128	0.2455	0.8021	0.57–1.13
*DRB4* *	81	111	0.1574	1.2960	0.92–1.81
*DRB5* *	25	61	0.0995	0.6430	0.40–1.05
*DQB1* * *02*	28	57	0.3957	0.7889	0.49–1.28
*DQB1* * *0301*	58	99	0.8942	0.9584	0.66–1.38
*DQB1* * *0302*	56	54	0.0027	1.9046	1.30–2.90
*DQB1* * *0303*	3	19	0.0320	0.2523	0.07–0.90
*DQB1* * *04*	19	47	0.1502	0.6419	0.37–1.12
*DQB1* * *05*	44	49	0.0529	1.5796	1.02–2.45
*DQB1* * *06*	32	72	0.1405	0.6973	0.44–1.10

* χ^2^test, significance level *p*<0.05.

**Table 3:** HLA Class II genes and rheumatoid arthritis patient characteristics.

RA features	HLADRB1*04	HLADBR1*04	*p*	HLADRB1*10	HLADRB1*10	*P*
	Negative	Positive		Negative	Positive	
Age at Onset (1)	49.8 (±11.9)	47.3±12.7	0.255	48.5 (±12.4)	48.8 (±7,8)	0.936
Positive RF^2^	36.5%	38.9%	0.228	71.6%	3.73%	0.193
Smoke (2)	16.4%	17.2%	0.668	31.3%	2.2%	0.202
Extra-articular manifestations (2)	14.9%	10.5%	0.322	23.9%	1.5%	0.444
UseAnti-TNF^2^	20.9%	16.4%	0.421	36.6%	0.7%	0.415

(1) Two-tailed Student *t* test, significance level *p*<0.05.

(2) χ^2^test for association, significance level *p*<0.05.

### A stepwise logistic regression model for HLA genes and RA

**Figure 1:**
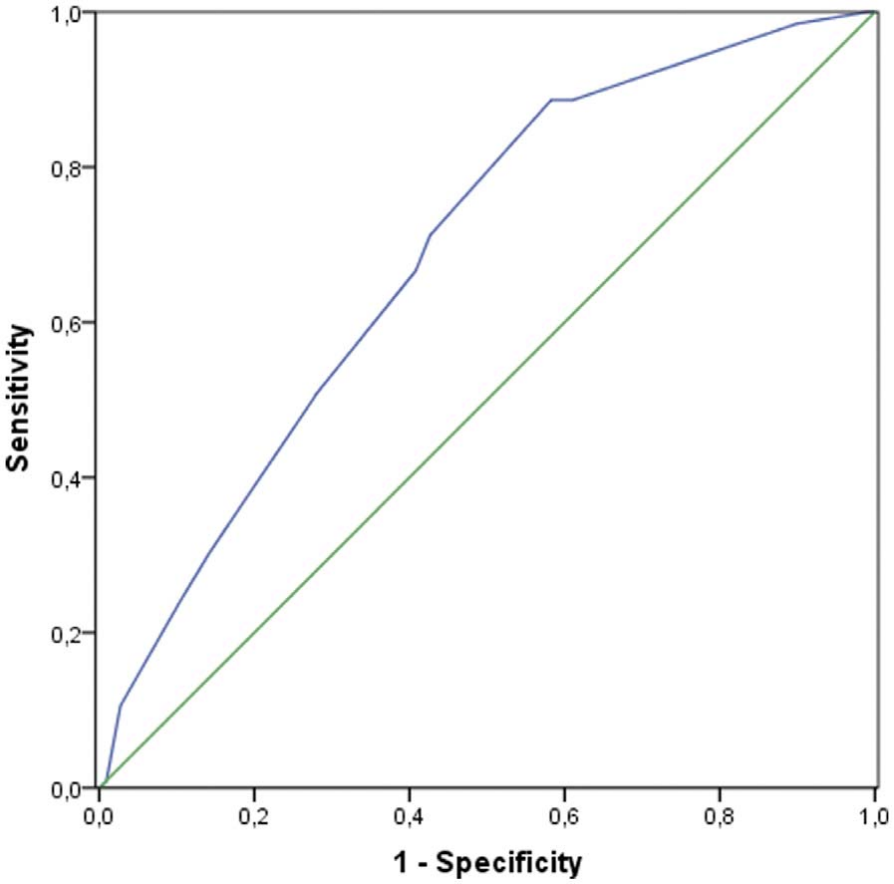
ROC curve for the general genetic model for RA susceptibility (AUC 0.70).

A general model of genetic risk for all HLA class II genes associated with RA based on logistic regression analysis (stepwise approach) is shown in Table 4. Genetic HLA data for both patients and controls were included in the statistical analysis. This analysis revealed that only HLA-DRB1*04, HLA-DRB1*16, HLA-DQB1*0302 and HLA-DQB1*05 fit the model. According to this regression analysis, HLA-DRB1*10, HLA-DQB*0303 and *HLA-DRB5* did not affect susceptibility to RA and were excluded from the final model. Again, *HLA-DRB1* was found to be an important factor in susceptibility to RA (*p* = 0.0001; OR 3.24). Figure 1 shows the ROC curves and the corresponding areas under the curves (AUCs), which indicate that accuracy of the model is acceptable (AUC≥0.7).

**Table 4:** Logistic Regression Model including Class II HLA for Rheumatoid Arthritis Patients^1^.

HLA genotype	*p*-value	OR	95% Confidence Interval
HLA-DRB1*04	0.0001	3.24	1.90–5.24
HLA-DRB1*16	0.0110	0.34	0.15–0.78
HLA-DQB1*0302	0.0360	1.71	1.03–2.81
HLA-DQB1*05	0.0100	2.37	1.40–4.02

1 Hosmer-Lemeshow test p = 0.485.

### The HLA-DRB1*04gene and high cardiovascular risk status in RA

**Figure 2:**
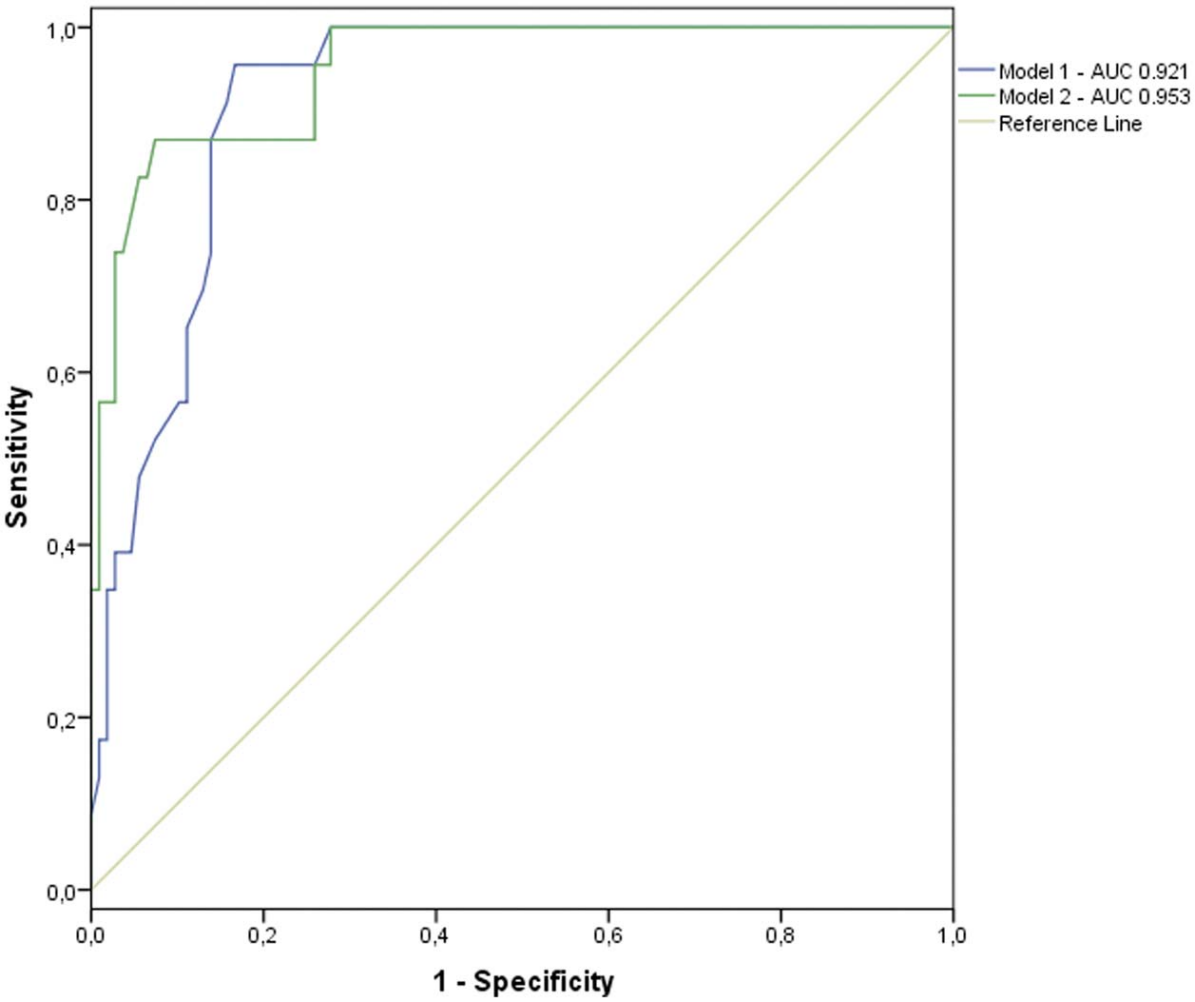
ROC curves comparing the accuracy of the two regression models. Smoking status is included in the second model.

**Figure 3:**
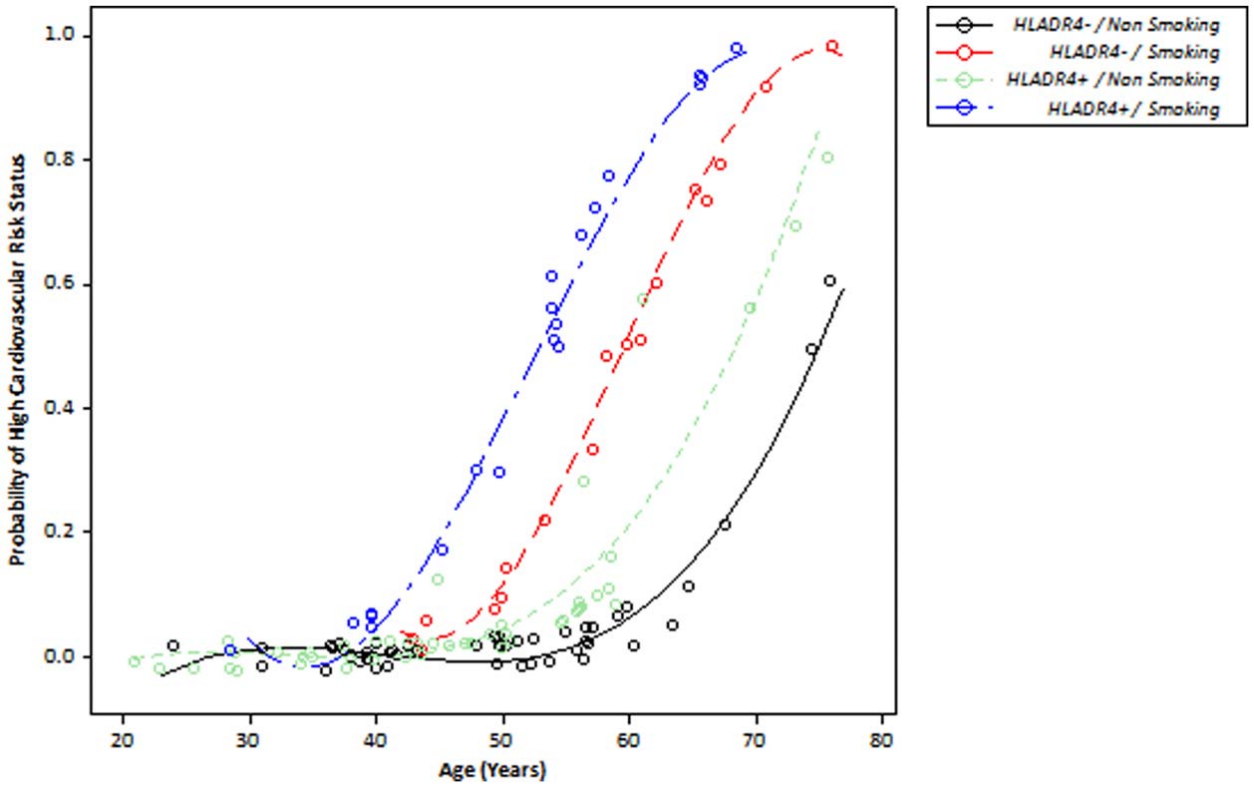
Event probability scatterplot for high cardiovascular risk status against age for different combinations of smoking status and presence/absence of the HLA-DRB1*04 gene.

High cardiovascular risk status is defined as a Framingham score of higher than 20%. RA patients were categorized as having a high or non-high cardiovascular risk. To analyze the influence of the HLA-DRB1*04 gene and smoking on cardiovascular risk status, the patients were included separately in two multivariate logistic regression models. First, the influence of the HLA-DBR1*04 gene on high cardiovascular risk status with age was observed in Model 1 (Table 5). A second logistic regression model including age, the HLA-DRB1*04 gene and smoking status was developed. This showed an association between smoking and HLADRB1*04 and high cardiovascular risk, with smoking raising the odds ratio of HLADRB1*04 to 6.02 and the *p*-value to 0.0270. Figure 2 shows the ROC curves and AUCs for the two models (AUC Model 1: 0.921; AUC Model 2: 0.953). The event probability scatter plot in Figure 3 shows the effect of the interaction between HLA-DRB1*04 and smoking status on high cardiovascular risk. Patients who smoke and carry the HLA-DRB1*04 gene appear to be at high cardiovascular risk earlier than smokers who do not carry the gene (Figure 3).

**Table 5:** Logistic Regression Models for High Cardiovascular Risk and HLA-DRB1*04.

Variable	*p*-value	OR	95% Confidence Interval
**First Model** (1)			
Age	0.0001	1.23	1.12–1.34
HLA-DRB1*04	0.0310	4.06	1.13–14.5
**Second Model** (2)			
Age	0.0001	1.26	1.13–1.39
Smoking	0.0001	23.3	4.27–32.1
HLA-DRB1*04	0.0270	6.02	1.21–29.7

(1) Hosmer-Lemeshow test, *p* = 0.962.

(2) Hosmer-Lemeshow test, *p* = 0.960.

## Discussion

RA is a chronic systemic autoimmune inflammatory disease that causes progressive articular damage. The severity of RA has been related to erosive disease and extra-articular symptoms, which compromise patient health and quality of life and reduce productivity [22], [23]. RA also has a major impact on life expectancy [24], which is about 3 to 10 years less for RA sufferers than for the general population [3]. Furthermore, the elevated mortality associated with RA has remained unchanged over the last two to three decades, and recent studies show that RA patients have not experienced the survival gains seen in the general population, so that the gap between the two populations has widened [25].

Patients with RA have a higher likelihood of developing comorbidities such as congestive heart failure, chronic pulmonary disease, dementia and peptic ulcer disease [26]. Over 50% of premature deaths in RA are due to cardiovascular diseases (CVD), including ischemic heart disease, congestive heart failure and stroke [27]. The mechanism behind this link between RA and CVD remains elusive. However, at least three domains appear to be important in this relationship: (1) traditional cardiac risk factors; (2) disease-related factors, such as elevated concentrations of inflammatory proteins or pro-inflammatory cytokines, genetic determinants and autoantibodies; and (3) treatment with drugs such as steroids, NSAIDS or cyclosporine [18].

A number of studies suggest that mortality due to CVD is substantially higher in individuals who are positive for rheumatoid factor (RF) and/or anti-citrullinated protein antibody (ACPA) and, most importantly, carry certain alleles of the *HLA-DRB1* gene [6], [11], [28]. Among the genetic risk factors for RA, both linkage and association studies of the *HLA-DRB1* locus have consistently confirmed it to be the major genetic susceptibility locus for RA [29].

A body of data has been collected about associations between HLA and RA in diverse ethnic populations, many of which display very different genetic backgrounds from those of Caucasians, in whom HLA-DRB1*0401 and HLA-DRB1*0404 are primarily associated with RA [30]. For example, in Japanese RA patients the allele associated with this disease is HLA-DRB1*0405 whereas in Chinese patients both HLA-DRB1*0405 and *0404 are associated with the disease. In some populations, such as the Spanish and Israelis, HLA-DRB1*04 alleles are rare [31]–[33]. In these settings, RA is associated with HLA-DRB1*01 or HLA-DRB1*10, two other alleles carrying the shared epitope [34]. It is interesting to note that we found that the rare allele HLA-DRB1*10 was associated with RA in the non-Indian Amazon population. This finding, which has only been reported in very few populations, has been described in the Portuguese population [35].

Although only Generic HLA Class II typing was performed in this study, our results clearly show that the HLA-DRB1*04 group is the main HLA type associated with susceptibility to RA in the study population. The results of other studies of the Brazilian population differ, however, and indicate that HLA-DRB1*0101 and DRB1*0102 were associated with susceptibility to RA whereas HLA-DRB1*0401 and DRB1*0404 were linked to more severe forms of the disease [36]. Interestingly, in Afro-Brazilian descendents HLADRB1*0404 and HLADRB1*0405 appear to be associated with susceptibility to RA [37]. These differences are probably related to a geographic variation in the genetic make up of the Brazilian population [38].

We also identified HLA-DRB1*16 as a protective allele for RA in our study population. It can thus be classified as a member of the S_3D_ group of alleles proposed by Tezenas du Montcel et al. [39] that confer protection against RA. These alleles (HLA-DRB1*1101, HLA-DRB1*1104, HLA-DRB1*12 and HLA-DRB1*16) are characterized by the DRRAA amino acid pattern at positions 70–74. This pattern in the third hypervariable chain of the HLA molecule is associated with a protective effect against RA [40].

We also found HLA-DQB1*0302 and HLA-DQB1*5 to be susceptibility genes for RA in our study population. While some association studies in humans have suggested that DQ alleles play a direct role in RA [40], [41], cumulative evidence indicates *DQB1* alleles have no influence on susceptibility to RA but that the clinical expression of the disease may be affected by DR–DQ complementarity [30].

Because of our relatively small number of patients, we did not investigate whether the *HLA-DRB1* genes influence RA severity as measured by classical radiology. Furthermore, we were unable to find any association between these genes and systemic manifestations of RA. High cardiovascular risk status is a consequence of the coexistence of several traditional cardiovascular risk factors such as smoking, hypertension, diabetes and advanced age and is implicated in an increased probability of a cardiac or cerebrovascular event within 10 years. In light of the impact and importance of CVD on the clinical and epidemiological features of RA, a high cardiovascular risk status must be considered when diagnosing and treating severe RA [42]. Thus, other non-traditional risk factors for cardiovascular events, such as the presence of the HLA-DRB1*04 allele, must be explored. Supporting this reasoning, our results showed that HLA-DRB1*04 influences the risk of CVD, increasing the probability of an individual with RA developing a high cardiovascular risk status at an early age. This effect is greater in HLA-DRB1*04 carriers who smoke than in those who do not.

It is important to note that CVD in RA may be affected by other genetic polymorphisms inside the MHC region such as *TNFA* rs1800629 (G>A), which seems to be restricted to individuals carrying the shared-epitope alleles associated with RA [43]. Other genetic polymorphisms outside the MHC region are also involved in cardiovascular events, showing that these events in RA are polygenic phenomena [44], [45].

The effect of the interaction between HLA-DRB1*04 and smoking on cardiovascular risk is probably not fortuitous. Modeling studies show that the shared epitope P4 pocket of the *HLA-DRB1* gene binds citrulline more effectively than arginine. When arginine residues were converted into citrulline by a citrullination process in RA, enhanced T-cell activation and increased binding of peptides by the shared epitope [46] were observed. It was then speculated that smoking leads to protein citrullination in the lungs and induces an exaggerated T-cell response in RA patients who smoke [47], [48]. This exaggerated T-cell response may be at least partly involved in the inflammatory endothelial process in arterial walls that sustain a pro-inflammatory response. In a recent study, interleukin-17 (IL-17), a T-cell derived cytokine, was the main predictor of microvascular function and arterial compliance, and the authors suggested that this cytokine may play a significant role in the development of endothelial dysfunction and CVD in RA and, also in pro-thrombotic phenotype, in human endothelial cells [49], [50].

Our study population included only patients from the Amazon Region in the north of Brazil. Nevertheless, several other studies, including one with Latin American patients, have found evidence that the *HLA-DRB1* gene, especially the*04 alleles, may play a role in endothelial activation, accelerated atherosclerosis and high mortality due to CVD [12], [51], [52]. Other evidence is emerging of an association between *HLA-DRB1* genotypes and early mortality. The Norfolk Arthritis Register (NOAR) suggests that carriage of two copies of shared-epitope alleles confers a higher risk of death from CVD [11]. Hence, it should be stressed that the data reported here confirm the association between HLA-DRB1*04 and cardiovascular disease risk in patients from the Brazilian Amazon. This finding was first reported and amply demonstrated in the European population by Spanish and UK researchers [11], [12], [52]. Moreover, our findings suggest that, while cardiovascular risk scores, such as the Framingham score, indicate which patients have a high ten-year cardiovascular risk, HLA-DRB1*04 may be useful for predicting which patients have a CVD phenotype at diagnosis and are therefore candidates for early, aggressive interventions. Nevertheless, further studies are necessary to clarify the role of HLA-DRB1*04 in the management of cardiovascular risk in patients with RA.

### Limitations

Some potential limitations of the study should be considered. As neither Afro-Brazilians nor Indians were included in the study, the *HLA* alleles that confer susceptibility to RA in these ethnic populations in the Amazon Region have yet to be identified. Furthermore, the relatively small number of patients included in the study may be an issue, given the frequency of RA in the Amazon Region. However, the results of the statistical analysis strongly supports the claim that HLA-DRB1*04 is associated with increased risk for cardiovascular disease in RA. In addition, high-resolution typing for HLA-DRB1*04 could have been performed to identify individual subtypes. In fact, some HLA-DRB1*04 allelic combinations include alleles encoding a shared epitope, such as HLA-DRB1*0401, *0404 and *0405, as well as others associated with low risk for RA such as *0402 and *0403. However, we can assume that in this case the association between HLA-DR4 and increased risk for cardiovascular disease in RA is mainly due to HLA-DRB1*04 and that shared epitope alleles are involved in susceptibility to RA.
